# Loaded n-Hydroxyapatite/SSG 3D Scaffolds as a Drug Delivery System of *Nigella sativa* Fractions for the Management of Local Antibacterial Infections

**DOI:** 10.3390/nano12050856

**Published:** 2022-03-03

**Authors:** Mohammed Dalli, Abdelqader El Guerraf, Salah-eddine Azizi, Karim Benataya, Ali Azghar, Jeong Mi-Kyung, Adil Maleb, Kim Bonglee, Nadia Gseyra

**Affiliations:** 1 Laboratory of Bioresources, Biotechnology, Ethnopharmacology and Health, Faculty of Sciences, University Mohammed the First, P.O. Box 524, Oujda 60000, Morocco; azizi.salah-eddine@ump.ac.ma (S.-e.A.); ngseyra@hotmail.com (N.G.); 2 Laboratory of Applied Chemistry and Environment, Faculty of Sciences, University Mohammed the First, P.O. Box 524, Oujda 60000, Morocco; elguerraf.chem@gmail.com (A.E.G.); k.benataya@ump.ac.ma (K.B.); 3 Laboratory of Microbiology, Hospital University Center/Faculty of Medicine and Pharmacy, P.O. Box 724, Oujda 60000, Morocco; aliazghar1992@gmail.com (A.A.); maleb.adil@gmail.com (A.M.); 4 KM Convergence Research Division, Korea Institute of Oriental Medicine, Yuseong-daero, Yuseong-gu, Daejeon 34054, Korea; oiny2000@kiom.re.kr; 5 Korean Medicine-Based Drug Repositioning Cancer Research Center, College of Korean Medicine, Kyung Hee University, Seoul 02447, Korea; 6 Department of Pathology, College of Korean Medicine, Kyung Hee University, Seoul 02447, Korea

**Keywords:** hydroxyapatite, 3D scaffolds, *Nigella sativa*, organic fractions, antibacterial activity

## Abstract

As a result of their close similarities to the inorganic mineral components of human bone, hydroxyapatite nanoparticles (n-HAp) are widely used in biomedical applications and for the elaboration of biocompatible scaffold drug delivery systems for bone tissue engineering. In this context, a new efficient and economic procedure was used for the consolidation of n-HAp in the presence of various *Nigella sativa* (*NS*) fractions at a near-room temperature. The research conducted in the present study focuses on the physicochemical properties of loaded n-HAp 3D scaffolds by *NS* fractions and the in vitro antibacterial activity against Gram-negative (*Escherichia coli* ATCC 25922, *Pseudomonas aeruginosa* ATCC 27853, *Klebsiella pneumoniae* ATCC 27853), and Gram-positive (*Staphylococcus aureus* ATCC 29213, *Enterococcus faecalis* ATCC 700603) bacteria. In order to better understand the effect of the inserted fractions on the HAp molecular structure, the elaborated samples were subject to Fourier transform infrared (FTIR) and X-ray diffraction (XRD) spectroscopic analyses. In addition, the morphological investigation by scanning electron microscope (SEM) of the loaded n-HAp 3D scaffolds demonstrated the presence of a porous structure, which is generally required in stimulating bone regeneration. Furthermore, the fabricated 3D composites exhibited significant antibacterial activity against all tested bacteria. Indeed, MIC values ranging from 5 mg/mL to 20 mg/mL were found for the HAp-Ethanol fraction (HAp-Et) and HAp-Hexane fraction (HAp-Hex), while the HAp-Aqueous fraction (HAp-Aq) and HAp-Methanol fraction (HAp-Me) showed values between 20 mg/mL and 30 mg/mL on the different strains. These results suggest that the HAp-*NS* scaffolds were effective as a drug delivery system and have very promising applications in bone tissue engineering.

## 1. Introduction

Calcium phosphate biomaterials are widely adopted in the reconstruction of large bone defects, which improves the health state of patients every day. Nano-crystalline hydroxyapatite (n-HAp: Ca_10_(PO_4_)_6_(OH)_2_) is one of the main components of human bones and constitutes up to 50% by volume, and 65% by weight, of bone [1,2]. This explains their suitable biological characteristics, including osteoinductivity and good bioactivity, larger surface area (100 m^2^ g^−1^), and biocompatibility with hard and soft tissues [3]. Additionally, n-HAp possess the ability to promote bone healing and induce cell viability and proliferation of mesenchymal stem (MSC) and osteosarcoma cells [4]. Furthermore, in vitro studies show that n-HAp can stimulate the osteogenic trans-differentiation of smooth muscle cells (SMCs) and support calcification on the extracellular matrix (ECM) [5]. What is more, n-HAp is used to inhibit proliferation and induce apoptosis in numerous cancer cells, including liver [6], breast [7], colon [8], and gastric cancer cells [9]. They are also commonly being used as carriers of various proteins, genes, drugs, anti-tumor agents, and other macromolecules [10,11]. Therefore, n-HAp is of interest in various biomedical fields, especially for bone tissue engineering and dental implant applications [12]. Thus, extensive research efforts have been conducted for the densification of n-HAp in order to produce bulk materials. In fact, the most adopted consolidation techniques are mainly carried out at very high temperatures (e.g., 1250 °C or higher). However, n-HAp is thermodynamically unstable at an extremely high sintering temperature, which influences, scientifically, the outstanding physicochemical and biological characteristics of the apatite phase, such as the low crystallinity and nanometric dimension of its particles [13]. Consequently, using such a conventional consolidation technique, the obtained materials are crystalline, with larger grain size and reduced in vivo reactivity. In our previous investigations, a novel approach at a near-room temperature was adopted to elaborate a highly biodegradable and biocompatible composite material based on n-HAp and sodium silicate glass (SSG) [14]. In this new method, a sodium silicate solution is used as a mineral binder to consolidate n-HAp while preserving their chemical and structural characteristics with a three-dimensional structure that possesses the adequate macro- and micro-porosities and mechanical profile that are required for bone-healing applications. The in vitro biodegradability and bioactivity tests demonstrated a fast reactivity of the elaborated scaffolds. In addition, the cytocompatibility tests assessed by cell viability and morphology proved that the n-HAp/SSG has a non-toxic effect and can show enhanced cell proliferation.

On the other hand, despite their good bioactivity, the n-HAp has no antibacterial properties, which restrict their clinical applications [15,16]. Additionally, various investigations found that bone tissue infections at implanted sites are usually associated with Gram-negative and Gram-positive bacteria, which can lead to serious complications after surgery [17,18]. Thus, a composite material with a chemical structure similar to the natural bone, in combination with antibacterial properties, is a highly demanded orthopedic biomaterial. Recent studies in the field have shown the use of hydroxyapatite as a local drug delivery system. Among them, we cite the study based on the absorption of the basilic and lavender essential oils on the hydroxyapatite surface that showed a good release of the tested essential oils and verified an important antibacterial activity [16]. In a similar vein, Huang et al. [19] described the antibacterial effect of chitosan-coated hydroxyapatite, and demonstrated the cytotoxic effect of the composite on normal cell lines.

*Nigella sativa* (NS), commonly known as black cumin, is a well-known plant with high distribution in Europe, the Middle East, Asia, and the north of Africa [20]. NS is found to be rich in different secondary metabolites such as polyphenols, flavonoids, tannins [21], and alkaloids [22,23]. It was also noted that black cumin has a large spectrum of pharmacological activities. It was found that the plant extracts exert an antibacterial [24], antifungal [25], antidiabetic [26], immunomodulatory [27], and analgesic effect [28].

The current study focuses on the antibacterial activity of n-HAp 3D scaffolds loaded with different NS fractions. This work combines the antibacterial activity exerted by the different fractions and the hydroxyapatite/SSG capacity to encapsulate the bioactive compounds present in each fraction. The morphology of the loaded n-HAp was observed using a scanning electron microscope (SEM), and the influence of the inserted fractions on the molecular structure of HAp was investigated by Fourier transform infrared (FTIR) and X-ray diffraction (XRD) spectroscopies.

## 2. Materials and Methods

### 2.1. Chemicals

Diammonium hydrogen phosphate ((NH_4_)_2_HPO_4_), calcium nitrate tetrahydrate (Ca(NO_3_)_2_·4H_2_O), ammonium hydroxide (NH_4_OH, 35%), sodium hydroxide (NaOH), methanol (CH_3_OH, ≥99.8%), ethanol (C_2_H_5_OH, ≥99.8%), dimethyl sulfoxide (C_2_H_6_O_S_, 99.9%) and hexane (C_6_H_14_, ≥97.0%) were purchased from Sigma-Aldrich (St. Louis, MO, USA) and used as received. Silica gel (SiO_2_, high-purity grades (Davisil Grade 633)) was also purchased from Sigma-Aldrich and used to prepare the sodium silicate solution. All solutions were prepared using highly purified water.

### 2.2. Plant Material

The *Nigella sativa* seeds were purchased and identified by professional botanists. A voucher specimen was deposited at the herbarium of the faculty under the number (HUMPOM471).

### 2.3. Preparation of Nigella sativa Fractions

**Figure 1:**
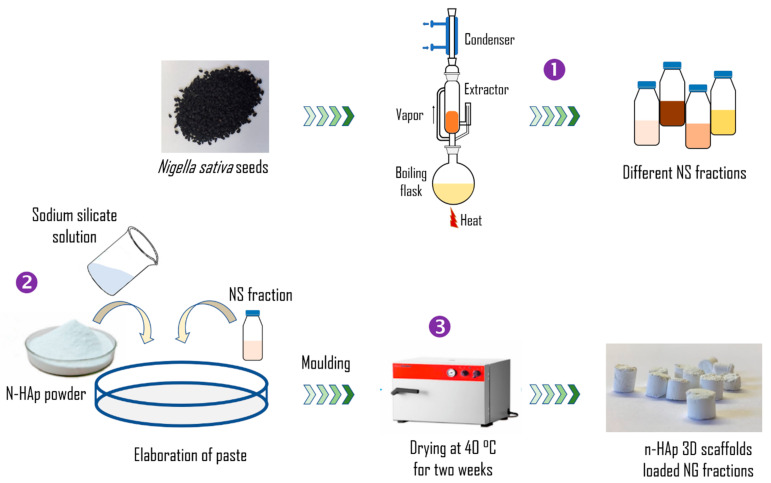
Schematic representation of the NS extraction method and 3D n-HAp-based composite scaffold fabrication. (1) extraction, (2) scaffolds preparation and (3) drying.

The different *Nigella sativa* seeds were taken and turned into fine powder, then 100 g of that powder was taken and put in a Soxhlet apparatus formed by an extractor, fitted in between a round-bottom flask at the bottom and a condenser at the top. Inside the extractor glass, the NS powder was placed within the thimble. The n-hexane was used for degreasing the plant. After that, the residual plant was extracted using ethanol. The remaining plant material was airdried again, and the extraction was assessed by methanol and water by following the same procedure after each extraction (Figure 1, step 1). The yields obtained ranged from 2.53% to 21.51%. The different obtained fractions were collected and stored at 4 °C for further use.

### 2.4. Synthesis of Hydroxyapatite Nanoparticles and Sodium Silicate Solution

HAp nanoparticles were prepared according to the method described in our previous work [14]. At room temperature, an aqueous solution of 0.20 M diammonium hydrogen phosphate was added drop-wise to a solution of 0.3 M calcium nitrate. The pH was maintained at 10 by adding 0.5 M ammonium hydroxide solution, and the mixture was kept in maturation for 30 min. Afterward, the precipitated n-HAp was filtered, washed with distilled water, and dried at 80 °C for 24 h.

A sodium silicate solution (Na_2_SiO_3_, H_2_O) with a molar ratio of SiO_2_/Na_2_O = 1 was prepared at 90 °C by dissolving pure NaOH pellets in distilled water, then an adequate amount of SiO_2_ was added to obtain a liquid glass containing, by weight, 25% SiO_2_, 25% Na_2_O, and 50% water.

### 2.5. n-HAp 3D Scaffolds Preparation

After the preparation of n-HAp, it was directly mixed with the sodium silicate solution and different NS fractions at a fixed liquid-solid ratio until a homogenous paste was obtained (Figure 1, step 2). The portion of n-HAp, sodium silicate solution and NS fractions in the prepared scaffolds were 70 wt%, 25 wt% and 5 wt%, respectively.

The malleable pastes were molded and oven-dried at 40 °C for 2 weeks to obtain dried bulk n-HAp/SSG/fractions composites (Figure 1, step 3).

### 2.6. Characterization

#### 2.6.1. XRD

The phases of the consolidated composites with different fractions were characterized through X-ray diffraction (XRD, XRD-6000, SHIMADZU, Kyoto, Japan) in the range of 2θ, an angle of 10–65° using Cu Kα radiation (λ = 0.154 nm), and a scanning speed of 4°/s. The crystallite size of the (002) plane was estimated by the Scherrer formula, as follows:

(1) $$D=\frac{K\lambda }{\beta \;\cos\theta }$$

D represents the crystallite size (nm); K is the Scherrer constant (k = 0.89); β is the half-width height of the diffraction peak; and θ is the diffraction angle of the associated (hkl) plane.

#### 2.6.2. FTIR

Fourier-transform infrared (FTIR) analysis in attenuated total reflection (ATR) mode is used for complementary structure identification. The experiment was conducted using a Jasco 4700-ATR spectrophotometer in the spectral range between 4000 and 500 cm^−1^ with a 4 cm^−1^ resolution.

#### 2.6.3. SEM

The microstructure of elaborated loaded composites was observed by scanning electron microscopy (FE-SEM) using a JEOL-JSM7001F apparatus. The working distance was maintained at 6 mm (the distance between the sample and the objective lens remains) and the SEM filament was operated at variable currents and a voltage of 5 kV using different magnifications. The elemental composition was determined using an X-ray energy dispersive spectroscopy (EDS).

### 2.7. Bacterial Strains

The bacterial strains used in the test were Staphylococcus aureus ATCC 29213 and Enterococcus faecalis ATCC 700603 (Gram-positive), and Escherichia coli ATCC 25922, Pseudomonas aeruginosa ATCC 27853, and *Klebsiella pneumoniae* ATCC 27853 (Gram-negative) were purchased from Thermoscientific.

### 2.8. Agar Diffusion Method

For the determination of the antibacterial activity of the different HAp 3D scaffolds, the agar diffusion method was adopted. All strains’ turbidity was normalized to 0.5 McFarland and then inoculated on the surface of the Petri dish. The HAp 3D scaffolds, loaded with different fractions, were placed on the surface of the Mueller–Hinton Agar (MHA). After 24 h of incubation at 37 °C, the inhibition diameter was measured. All measurements of inhibition zones were performed in triplicate.

### 2.9. Minimal Inhibitory and Minimal Bactericidal Concentration

The microdilution technique was used for the determination of the minimal inhibitory concentration [29]. The different tested fractions were dissolved in 2% DMSO, and then a serial dilution was prepared by Mueller–Hinton Broth. A total of 180 µL of each concentration was taken and added to each well. After that, 20 µL of the bacterial suspension was added to all wells. All plates were then incubated at 37 °C for about 24 h. To facilitate the determination of the MIC value, the resazurin, an indicator of bacterial growth, was added to the mixture (10 µL) and the plates were incubated for an additional 2 h. The well with no color change was considered as the MIC value.

For the determination of the minimal bactericidal concentration, 20 µL was taken from each well with no color change and inoculated on the surface of the Petri dish, and then incubated at 37 °C for about 24 h. The dish with no subculture was considered as the MBC. All tests were performed in triplicate.

## 3. Results and Discussion

### 3.1. Physicochemical Characterization of the Composite Scaffold

**Figure 2:**
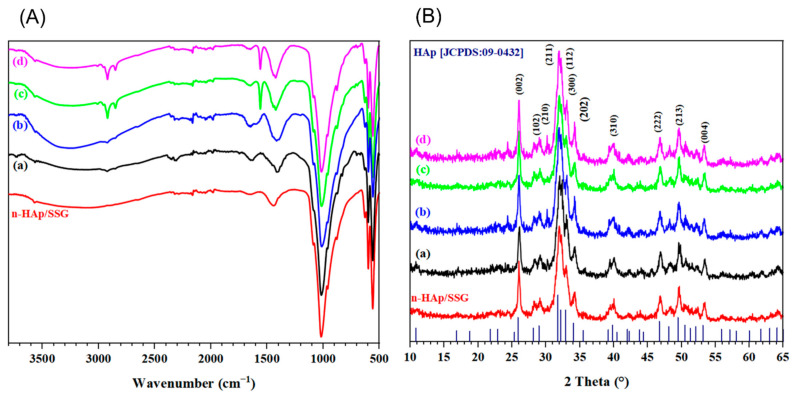
ATR-FTIR (**A**) and XRD patterns (**B**) of consolidated samples. (a) HAp-Aq, (b) HAp-Me, (c) HAp-Et, and (d) HAp-Hex.

Consolidated composite materials are characterized by ATR-FTIR and depicted in Figure 2A. The ATR-FTIR spectra highlight that all consolidated materials present characteristic vibrational bands of phosphate groups (PO_4_^3−^) of n-HAp structure, specifically at nearly 570, 603.7 cm^−1^, 960 cm^−1^, and between 1010 and 1110 cm^−1^. The two vibrational bands observed at 630 and 3540 cm^−1^ are assigned to the hydroxyl group (O–H) of the n-HAp structure. The small band around 1420 cm^−1^ in the pure composite material n-HAp/SSG is attributed to the carbonate ions CO_3_^2−^ [30]. The intensity of this band increased as the fractions were incorporated in the materials. Additionally, two more peaks appeared at approximately 1640 cm^−1^, and between 2700 and 3200 cm^−1^, corresponding to the functional groups present in the organic compounds in the *NS* fractions. The sharp band at 1640 cm^−1^ can be assigned to the C=C stretching vibration [16]. In the 2700–3200 cm^−1^ spectral region, the bands situated between 2700 and 3200 cm^−1^ are attributed to the C–H stretching vibrations [31].

The structure and phase composition of the consolidated materials loaded are investigated as well, using XRD analysis (Figure 2B). As observed in the figure, the XRD patterns of all elaborated samples exhibited peaks with high-intensity characteristics that are in good agreement with JCPDS data for HAp (JCPDS file No. 09-0432). Additionally, the broadened peaks indicate the low crystallinity of the apatitic phase in the elaborated materials. Based on the XRD diffraction peak (002), the crystallite size of n-HAp is determined to be in the range of 37 to 40 nm for non-loaded and loaded scaffolds, respectively, which illustrates the nanocrystalline feature of the consolidated HAp phases. These results suggest that 3D composites are mainly formed by an apatitic phase with low crystallinity and nanoparticles similar to the inorganic phase of young bones (newly formed bone tissues) [32].

**Figure 3:**
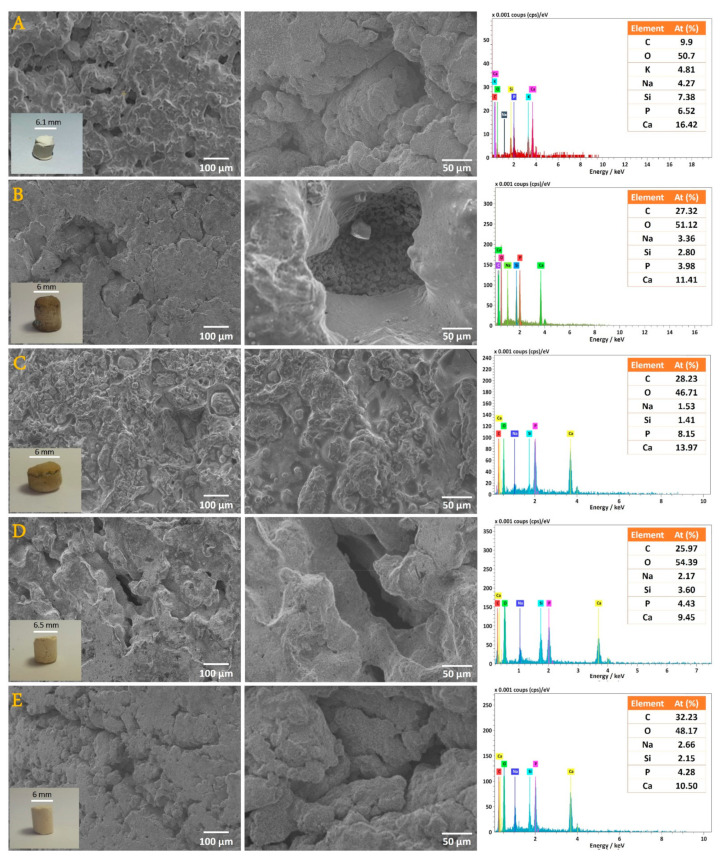
SEM micrographs and the corresponding EDS patterns of consolidated samples. (**A**) HAp/SSG, (**B**) HAp-Aq, (**C**) HAp-Me, (**D**) HAp-Et and (**E**) HAp-Hex.

On the other hand, SEM investigation was achieved to describe the resulting structure of the 3D composites. The micrographs of various NS fraction-loaded composites are presented in Figure 3. For comparison, the analysis was also achieved for non-loaded n-HAp/SSG and the images are gathered in the same figure. As shown, the morphology of n-HAp is affected by the introduced molecules. Indeed, each NS fraction interacts differently with HAp, giving various topographies.

**Figure 4:**
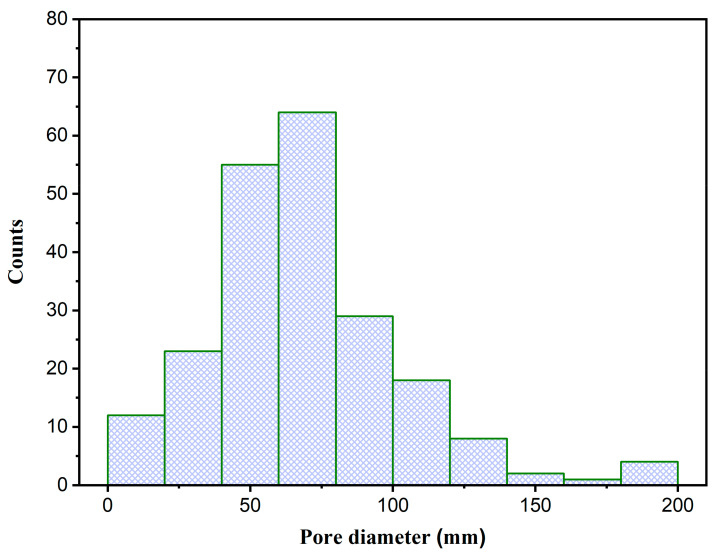
Statistical histogram of the pore size distribution derived from the SEM micrographs.

The SEM revealed a compact and homogeneous structure where n-HAp particles are totally integrated within the SSG material (Figure 3A). Hence, this results in the formation of a high number of pores spread equally throughout the 3D surface. After the modification of n-HAp/SSG, each introduced NS fraction presents a specific morphology. It can be seen that the fraction prepared with methanol solvent (HAp-Me) can block some of the pores. As shown, a smooth coating layer has formed, which covers the entire outer surface of n-HAp/SSG (Figure 3C). However, many micropores with a diameter generally lower than 50 μm are still present. On the other hand, the aqueous-, ethanol-, and hexane-based fractions demonstrate the presence of randomly distributed and interconnected micro-porosity (Figure 3B,D,E). To obtain a comprehensive understanding of the pore space of the samples under investigation, the histogram of the pore size distribution, plotted using processed SEM micrographs from ImageJ, is presented in Figure 4. The specimens display a unimodal distribution with a mean pore size of 68.63 μm. In fact, the pore diameters ranged between 10 and 200 μm for practically all elaborated loaded scaffolds.

Commonly, the presence of abundant irregular open and interconnected pores on the outer and inner surfaces of a biomaterial is an important key for industrial applications. This interesting property has a crucial role in stimulating bone regeneration, as it provides a great ability to induce osteointegration and improve implant adhesion to the host tissue.

The EDS analysis of the NS-based composite scaffolds, presented in the same figure, clearly indicates the presence of calcium (Ca) and phosphorus (P) from the n-HAp particles, and silicon (Si) and sodium (Na) resulted from the precipitated sodium silicate glass. Additionally, carbon (C) and oxygen (O) can be associated with either n-HAp or SSG. By comparison to the atomic percentage of carbon in n-HAp/SSG before and after the integration of NS fractions, we note an important increase in the organic matter in our composites. Indeed, C% of about 9.9% was obtained for the unmodified composite (as shown in Figure 3 from our previous paper [14]); it passes to 27%, 28%, 25%, and 32% for HAp-Aq, HAp-Me, HAp-Et, and HAp-Hex, respectively.

### 3.2. Antibacterial Activity

The antibacterial susceptibility testing of the 3D HAp scaffolds loaded with different *NS* fractions was assessed on the following Gram-positive and Gram-negative bacterial strains: *Staphylococcus aureus* ATCC 29213, *E. coli* ATCC 25922, *Pseudomonas aeruginosa* ATCC 27853, *Klebsiella pneumoniae* ATCC 27853, and *Enterococcus faecalis* ATCC 700603. The results obtained using the agar diffusion method are given in Table 1.

The agar diffusion method was used for testing the antibacterial capacity of the different *NS* fractions. The results obtained for the 3D HAp scaffold-free fractions demonstrate no activity on the tested strains, while loading the 3D scaffolds with aqueous, methanol, ethanol, and n-hexane fractions showed that all strains were sensitive toward all n-HAp scaffolds. Due to the fact that the HAp 3D scaffolds free of any additional fraction have no effect, the antibacterial activity was attributed to the *NS* fractions.

Concerning the MIC and MBC values, the results are depicted in Table 2. The different MIC values were ranging from 5 to 20 mg/mL, while the MBC values were from 5 to 50 mg/mL.

**Figure 5:**
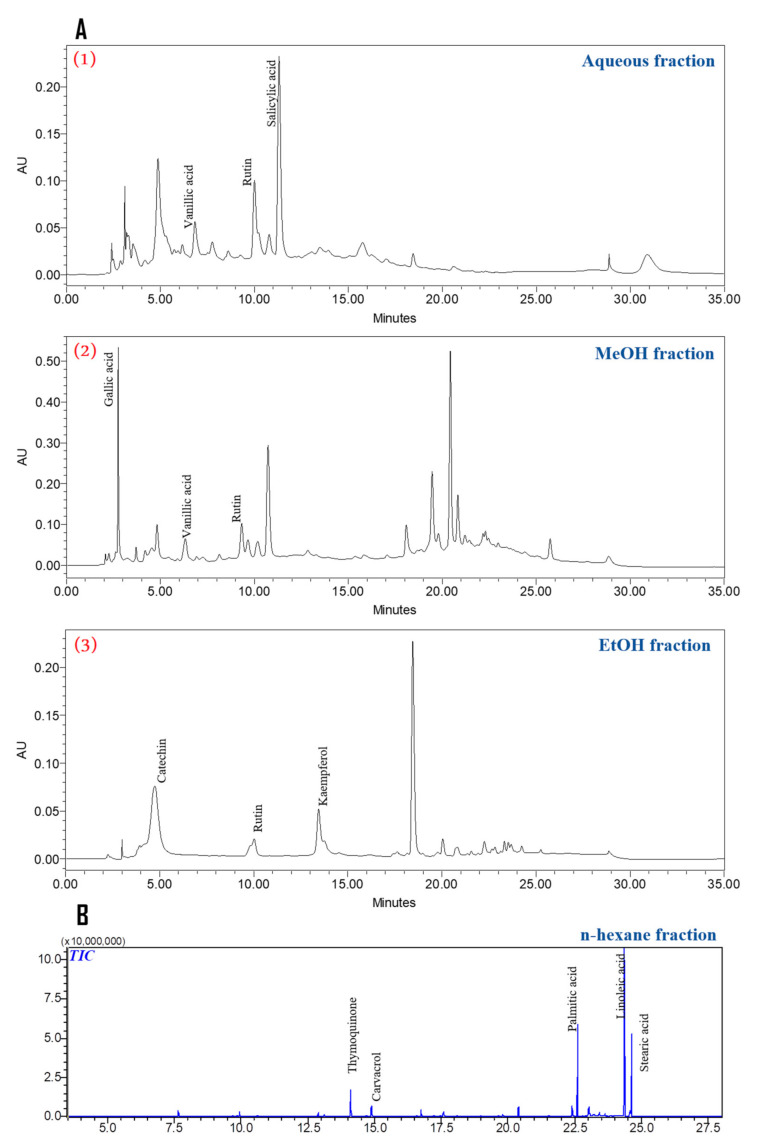
HPLC profiles of the different tested fractions (**A**) and the total ion chromatogram of the n-hexane fraction (**B**). (1) aqueous fraction, (2) methanol fraction and (3) ethanol fraction.

The present study aimed to investigate the antibacterial activity of different n-HAp-loaded scaffolds with different NS fractions obtained from Soxhlet apparatus by using a new consolidation technique. According to our previous studies, it was mentioned that the *NS* seeds are of great richness with phenolic and flavonoid compounds [21]. For example, the aqueous fraction was found to be rich in vanillic acid, ferulic acid, salicylic acid, vanillin, and rutin (Figure 5A(1)). In addition, the MeOH fraction was characterized by the presence of gallic acid, vanillic acid, rutin, and naringenin (Figure 5A(2)), while the EtOH fraction was rich with three flavonoids (rutin, catechin, kaempferol) (Figure 5A(3)). On the other hand, the n-hexane methyl ester was distinguished by the presence of thymoquinone, linoleic acid, palmitic acid, carvacrol, and stearic acid, with different amounts (Figure 5B) [26].

Regarding the antibacterial activity, the results obtained indicate that the different tested fractions are endowed with a strong antibacterial activity that could be attributed to the bioactive compounds present in each fraction. The smallest MIC value was registered in Gram-positive bacteria (*S. aureus* and *E. faecalis*) with a value of 5 mg/mL in HAp-Hex and HAp-Et. The highest MIC value was 30 mg/mL for *E. faecalis* and 20 mg/mL for the other bacteria. From the results, it was observed that the Gram-positive bacteria were very sensitive to the n-HAp-Hex and n-HAp-Et, while the Gram-negative bacteria were less sensitive to the tested fractions. This difference in response between the Gram-positive bacteria and the Gram-negative bacteria is principally due to the morphology of the bacterial wall. The Gram-positive bacteria are characterized by their richness with peptidoglycans, which facilitate the passage of hydrophobic molecules to the inside of the bacteria. The Gram-negative bacteria wall is rich with lipopolysaccharides, which allow the passage of the hydrophilic molecules from the external environment to the internal environment [33].

In a study performed on the methanolic extract of NS, it was noted that the extract exerts a strong activity on *S. aureus*, while it was inactive on *K. pneumoniae*. In the same context, Zuridah et al. [34] demonstrated a weak activity on *E. coli* and *P. aeruginosa*. These findings were different when compared with our results, where the MeOH fraction was able to inhibit the *K. pneumoniae*, *E. coli*, and *P. aeruginosa* at an MIC value of 20 mg/mL.

Regarding the black cumin ethanolic extract tested on bacteria that alter food, it was shown that this extract inhibits the bacteria *S. aureus* and *E. coli*, with no effect on *K. pneumoniae* growth [35], which was contradictory to our findings, where the EtOH fraction showed a great inhibitory potential toward all strains tested with an MIC value ranging from 5 to 20 mg/mL. The study of Khalid et al. [36] demonstrated that the aqueous extract has a weak antibacterial activity toward *S. aureus*, *E. coli*, *P. aeruginosa*, and *E. faecalis*, while in the present study it was demonstrated that all tested strains manifested a high sensitivity to the NS aqueous fraction. Concerning the n-hexane fraction, the MIC values ranged from 5 to 20 mg/mL, which was close to those obtained in the Abraham et al. study [37].

Several studies have reported the antibacterial potential of the different molecules identified in our fractions. For example, the salicylic acid present in our aqueous fraction was found to be endowed with an important inhibitory activity on different bacteria such as *S. aureus*, *E. coli*, *P. aeruginosa*, and *E. faecalis* with an MIC (250–500 µg/mL). In the same study, it was registered that the quercetin present in our MeOH fraction, and the kaempferol present in our EtOH and aqueous fractions, were found to play a moderate antibacterial role with an MIC value of about 500–1000 µg/mL. Moreover, the rutin was found to be capable of inhibiting the different strains tested with an MIC ranging between 500 and 1000 µg/mL [38]. The two phenolic acids (gallic and ferulic acids) were reported to exhibit an important antibacterial activity on different bacteria such as *S. aureus*, *E. coli*, and *P. aeruginosa*, with an MIC value between 100 and 1750 µg/mL. It was also noted that exposure to gallic acid and ferulic acid induced membrane damage in the tested bacterial strains [39]. The catechin present in our MeOH, EtOH, and aqueous fractions was revealed to have an important antibacterial activity characterized by creating damage to the cell membrane [40]. On the other hand, it was noted that long-chain unsaturated fatty acids are capable of inhibiting Gram + and Gram—bacteria [41], while TQ present in the n-hexane fraction was mentioned to be endowed with a great antibacterial activity, especially on multidrug-resistant bacteria [42]. It was also mentioned that carvacrol causes an alteration in the fluidity and permeability of the membrane, which is attributed to the hydroxyl group present on the molecule. In addition, this terpenoid compound could cause a leakage of K^+^ ions and an exhaustion of intracellular ATP, which explains the various alterations induced [43,44]. Likewise, the carvacrol was found to be capable of blocking the flagellins, which induces a cessation of bacterial motility [45], and it was registered that the linoleic acid has an antibacterial activity, especially on methicillin-resistant staphylococcus aureus, which could be used as a therapeutic agent. Finally, in a previous study, the different fractions were found to be non-toxic, which indicates that these fractions are free of any hazardous effects [26].

**Figure 6:**
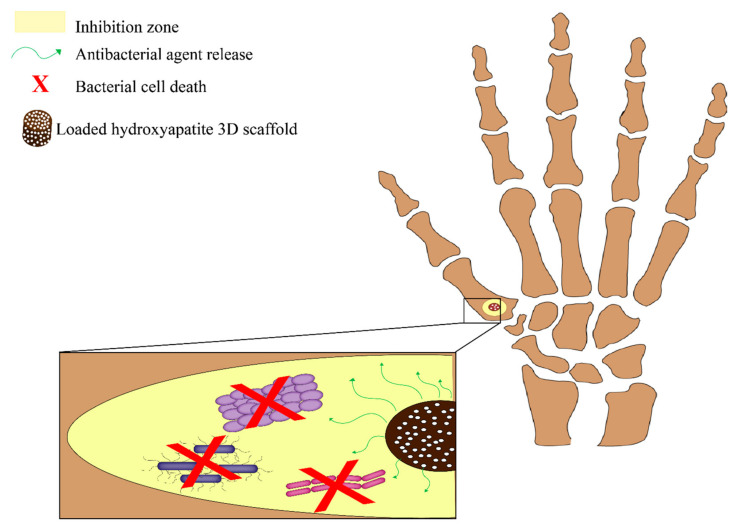
Schematic representation of the loaded hydroxyapatite/SSG 3D scaffold as a promising biomaterial for orthopedic applications.

In summary, the synthesized 3D scaffolds based on HAp and NS fractions exhibited interesting antibacterial activities due to the interaction of various bioactive compounds present in each fraction with the outer membrane of the bacteria. The amelioration of n-HAp biological properties by loading with an NS fraction has very promising clinical applications that can eliminate any bone tissue infections (Figure 6).

## 4. Conclusions and Future Perspectives

In this paper, hydroxyapatite nanoparticles were successfully consolidated in the presence of various NS fractions. The findings reveal that the NS exerted antibacterial activity on several Gram-positive and Gram-negative bacteria that were loaded on hydroxyapatite 3D scaffolds as a drug delivery system. This effect could be attributed to the different bioactive compounds identified in the tested fractions, such as gallic acid, salicylic acid, vanillic acid, rutin, catechin, kaempferol, thymoquinone, linoleic acid, and carvacrol. These obtained results could be considered a significant contribution to the development of new applications in the medical field. The use of these bioactive compounds loaded in hydroxyapatite/SSG composite in bone reconstruction could help in the amelioration of the health state by reducing the number of postoperative infections after the implants. More studies are in progress in order to investigate, in vivo, the capacity of the loaded hydroxyapatite 3D scaffolds.

## Figures and Tables

**Table 1:** Inhibition zone diameters of *NS*-loaded fractions.

	*S. aureus* ATCC 29213	*E. faecalis* ATCC 25922	*P. aeruginosa* ATCC 27853	*K. pneumoniae* ATCC 27853	*E. coli* ATCC 700603
HAp/SSG	0	0	0	0	0
HAp-Aq	14.67 ± 0.26	9.50 ± 0.19	16.2 ± 0.13	15.67 ± 0.44	17 ± 0.45
HAp-Me	11.5 ± 0.26	11.17 ± 0.32	14.7 ± 0.25	14.83 ± 0.22	16.67 ± 0.26
HAp-Et	13.75 ± 0.19	14.50 ± 0.19	14 ± 0.001	14.17 ± 0.556	15.67 ± 0.26
HAp-Hex	11.17 ± 0.13	11 ± 0.38	16 ± 0.577	15 ± 0.7	15.50 ± 0.38

Inhibition diameter of the different tested strains (mm).

**Table 2:** MIC and MBC in mg/mL of *Nigella sativa*-loaded fractions in HAp 3D scaffolds against tested bacteria.

	*S. aureus* ATCC 29213		*E. faecalis* ATCC 25922		*P. aeruginosa* ATCC 27853		*K. pneumoniae* ATCC 27853		*E. coli* ATCC 700603	
	*MIC*	*MBC*	*MIC*	*MBC*	*MIC*	*MBC*	*MIC*	*MBC*	*MIC*	*MBC*
HAp-Aq	20	20	30	50	20	30	20	30	20	30
HAp-Me	20	20	20	30	20	30	20	30	20	30
HAp-Et	5	5	5	5	10	20	20	30	10	20
HAp-Hex	5	5	5	5	20	20	20	20	10	20

MIC and MBC are minimal inhibitory concentration and minimal bactericidal concentration, respectively.

## Data Availability

Data are available within the article.
